# Laser Microdissection-Based Tissue-Specific Transcriptome Analysis Reveals a Novel Regulatory Network of Genes Involved in Heat-Induced Grain Chalk in Rice Endosperm

**DOI:** 10.1093/pcp/pcy233

**Published:** 2018-12-04

**Authors:** Tsutomu Ishimaru, Sabiha Parween, Yuhi Saito, Takanari Shigemitsu, Hiromoto Yamakawa, Mikio Nakazono, Takehiro Masumura, Naoko K Nishizawa, Motohiko Kondo, Nese Sreenivasulu

**Affiliations:** 1 NARO Institute of Crop Science, NARO, 2-1-18 Kannondai, Tsukuba, Ibaraki, Japan; 2 Hokuriku Research Station, Central Region Agricultural Research Center, National Agriculture and Food Research Organization (CARC/NARO), 1-2-1 Inada, Joetsu, Niigata, Japan; 3 International Rice Research Institute (IRRI), DAPO, Metro Manila, The Philippines; 4 Graduate School of Life and Environmental Science, Kyoto Prefectural University, Shimogamo, Sakyo-ku, Kyoto, Japan; 5 Graduate School of Bioagricultural Sciences, Nagoya University, Furo, Chikusa, Nagoya, Japan; 6 Research Institute for Bioresources and Biotechnology, Ishikawa Prefectural University, 1-38 Suematsu, Nonoichi, Ishikawa, Japan

**Keywords:** Developing endosperm, Gene expression, Grain chalkiness, Heat stress, Laser-microdissection, *Oryza sativa*

## Abstract

Heat stress occurrence during seed filling leads to the formation of a chalky portion in the limited zone of the starchy endosperm of rice grains. In this study, isolation of aleurone, dorsal, central and lateral tissues of developing endosperm by laser-microdissection (LM) coupled with gene expression analysis of a 44 K microarray was performed to identify key regulatory genes involved in the formation of milky-white (MW) and white-back (WB) grains during heat stress. Gene regulatory network analysis classified the genes changed under heat stress into five modules. The most distinct expression pattern was observed in modules where most of the small heat shock proteins and cellular organization genes were changed under heat stress in dorsal aleurone cells and dorsal starchy endosperm zones. The histological observation supported the significant increase in cell number and size of dorsal aleurone cells in WB grains. With regard to the central starchy endosperm zone, preferential down-regulation of high molecular weight heat shock proteins (HMW HSPs), including a prominent member encoding endoplasmic reticulum (ER) chaperones, by heat stress was observed, while changes in expression of starch biosynthesis genes were minimal. Characterization of transgenic plants suppressing endosperm lumenal binding protein gene (*BiP1*), an ER chaperone preferentially down-regulated at the MW zone under heat stress, showed evidence of forming the chalky grains without disturbing the expression of starch biosynthesis genes. The present LM-based comprehensive expression analysis provides novel inferences that HMW HSPs play an important role in controlling redox, nitrogen and amino acid metabolism in endosperm leading to the formation of MW and WB chalky grains under heat stress.

## Introduction

The appearance of rice grains, such as the degree of translucency, is extremely important to capture higher revenues in the market. Chalky grains drastically increase when rice plants are subjected to a mean temperature >27�C during the first 20 d after heading (Wakamatsu et�al. 2007). A proportion of chalky grains >15% has a negative impact on eating quality (Kim et�al. 2000), due to the decrease in palatability of the rice grain (Kim et�al. 2000, Cheng et�al. 2005). In addition, the proportion of chalky grains is negatively correlated with head rice yield (Lyman et�al. 2013). Designing the development of climate-resilient rice with acceptable grain quality for heat stress is a pressing issue (Sreenivasulu et�al. 2015).

The grain chalk which occurs under heat stress is categorized into several major types. Milky-white (MW) and white-core (WC) types have chalk around the central zone of the starch endosperm, while white-back (WB) and basal-white (BW) types have chalk at the peripheral part of the starch endosperm (Morita et�al. 2016). Starch accumulation in the developing endosperm is asynchronous. Active accumulation of starch starts in the central zone at an early stage, then it proceeds to the peripheral parts as seed maturation progresses (Ishimaru et�al. 2009). The timing of heat exposure during onset of seed formation and grain filling causes differences in the types of chalky grains (Tashiro and Wardlaw 1991). Interstingly, even among tissues in starchy endosperm, the center and back zones are likely to be chalky, whereas a chalky phenotype is not observed at the lateral zone under high-temperature stress (Ishimaru et�al. 2009). In addition to the tissue-specific chalky phenotypes in starchy endosperm, aleurone layers seem to be affected by heat stress (Nagato and Ebata 1960). However, the tissue-specific alteration affecting individual tissues under heat stress causing different chalk phenotypes is unknown and thus its association with molecular mechanisms needs to be investigated.

Among the reported starch-deficient mutants, differences in amyloplast development were shown to impact grain appearance between perfect translucent vs. opaque chalky grain. In the perfect grains, amyloplasts were regularly packed with more uniform starch granules without any air gaps. Under heat stress, amyloplasts are loosely packed with different shapes and sizes in the chalky part of the grains (Tashiro and Wardlaw 1991, Yamakawa et�al. 2007, Ishimaru et�al. 2009). The development of amyloplasts is aberrant during grain filling in the chalky parts. Hakata et�al. (2012) demonstrated that suppression of α-amylase genes during seed filling confers heat tolerance by producing a lower percentage of chalky grains. Some of key genes related to the formation of chalky grains under heat stress have been identified from the pathway of starch metabolism and catabolism enzymes (for a review, see Mitsui et�al. 2016). To date, we lack understanding of the effect of high temperature treatment on tissue-specific amyloplast development and differentiation in central and dorsal zones of starchy endosperm.

High temperature has been shown to impact proline accumulation during seed filling, leading to an effect on the chalky appearance and grain quality (Lin et al. 2010). High temperature induces the expression of heat shock proteins (HSPs) in developing rice grains (Lin et�al. 2005, Yamakawa et�al. 2007, Liao et�al. 2014), possibly to stabilize the function of target proteins by folding them under stressful environmental conditions as a molecular chaperone. Recently, genetic studies on HSPs have elucidated the involvement of altered expression of HSPs on grain chalk phenotype in rice. The artificial suppression of endosperm lumenal binding protein (BiP; *BiP1*) and protein disulfide isomerase (PDI; *PDIL1-1*), key chaperones involved in folding of secretory seed storage protein also leads to the chalky phenotype (Wakasa et�al. 2011, Kim et�al. 2012). In the notched-belly mutant with the white-belly type of chalky grains, *BiP1* and an isoform of PDI (*PDIL2-3*) were down-regulated (Lin et�al. 2014). A mutant defective in *flo11* encoding a plastid-localized HSP 70 (OsHsp70cp-2) causes the chalky grain phenotype through impaired amyloplast development (Zhu et�al. 2018). These pieces of evidence imply the highly complex molecular mechanism with regards to the involvement of organelle-localized HSPs, which is not directly related to the carbohydrate-metabolizing pathway, but affects organelle development and altered storage processes in rice endosperm development. However, the effects of changes in expression of HSPs on heat-induced chalky grains (i.e. MW and WB grains) in distinct seed tissues have not been investigated yet.

Laser-microdissection (LM) is a powerful tool for isolating targeted individual cells from heterogeneous tissue viewed under a microscope, using an intense laser beam (Emmert-Buck et�al. 1996). To date, the LM technique has been applied to a number of plant organs to investigate the global expression of genes in the target tissue (for a review, see Sreenivasulu and Wobus 2013). However, LM has not yet been applied to the stress physiology of developing cereal seeds, which is a critical issue in the grain quality to unravel molecular mechanisms influencing distinct chalky phenotypes under heat stress. We previously developed an LM-based method for obtaining high-quality RNA from developing rice endosperm, facilitating precise expression analysis of specific tissues (Ishimaru et�al. 2007). The LM technologies coupled with comprehensive expression analysis would provide a novel break-through to unveil the complex molecular basis of formation of each type of grain chalk through changes in the expression level of genes in distinct tissue types of starchy endosperm and aleurone cells greatly affected by heat stress.

In the present study, different zones of developing endosperm, which show contrasting chalk phenotypes under heat stress, were isolated with LM, and the 44 K Agilent microarray system was employed to compare the changes in gene expression in each tissue between control and high-temperature treatments. The aim of this study is to reveal the novel pathways and regulatory network from tissue-specific changes in gene expression of MW and WB types of grain chalk induced by heat stress.

## Results

### Histological changes of rice grain in control and high-temperature conditions

Under control conditions, the grain appearance was translucent in the entire grain (Fig.�1A) and endosperm growth is normal, as evidenced in the transversal section (Fig.�1B, C). In contrast, the appearance of grains with MW + WB types of chalk looked entirely chalky under high-temperature conditions (Fig.�1D). Transversal sections of MW + WB types of grain revealed that the chalky phenotype in the dorsal zone extended to the central zone of starchy endosperm, while the aleurone layer and lateral zones were not chalky (Fig.�1E, F). Scanning electron microscopy (SEM) observation showed regularly shaped amyloplasts, which were tightly packed in the translucent parts of perfect grains in control conditions (Supplementary Fig. S1A). In contrast, irregularly shaped amyloplasts were loosely packed at central (Supplementary Fig. S1B) and dorsal (Supplementary Fig. S1C) zones of MW and WB grains, respectively, under high-temperature conditions. In this study, dorsal aleurone cells, and starchy endosperm at the dorsal, central and lateral zones are abbreviated as AL, DSE, CSE and LSE, respectively (Fig.�1B, E, F).

**Fig. 1:**
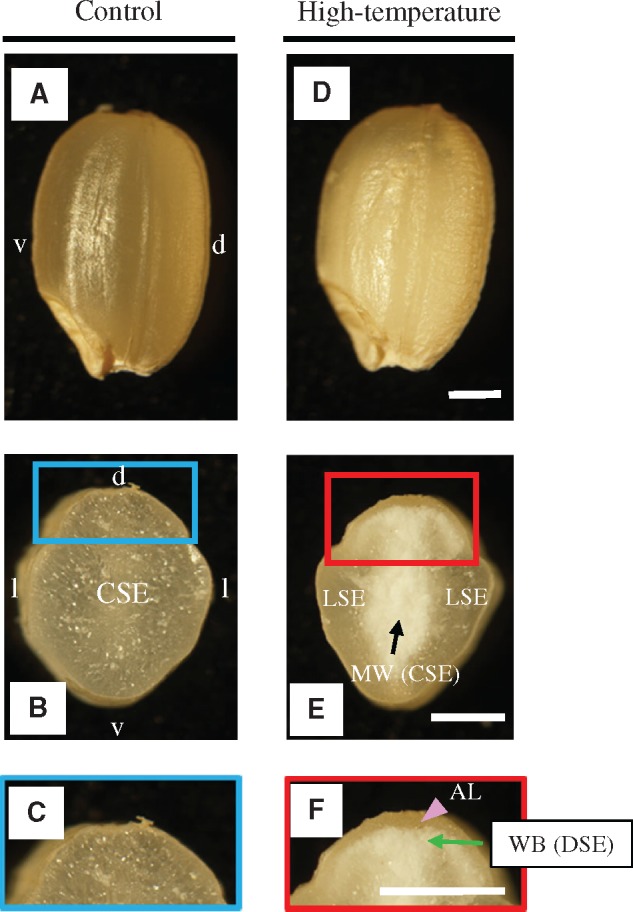
Typical grain phenotypes observed in control (A–C) and high-temperature (D–F) conditions. Appearance of a perfect grain from control treatment (A) and a chalky grain from high-temperature treatment (D). d, dorsal side; v, ventral side. Median transverse section of a perfect (B) and a chalky (E) grain. The cental starchy endosperm (CSE) and lateral starchy endosperm (LSE). l, lateral side; the CSE zone corresponds to the milky-white (MW) type of chalk in high-temperature conditions. Magnified scale of the dorsal side in control (C) and high-temperature (F) conditions. The dorsal aleurone layer (AL) and dorsal starchy endosperm (DSE). The DSE zone corresponds to the white-back (WB) type of chalk in high-temperature conditions. Abbreviations are also applicable to the subsequent figures and the tables. Scale bars = 10 mm.

### Frequency of chalky grains by temporal exposure to high temperature during ripening

Exposure to high temperature during the entire storage phase [4–35 days after flowering (DAF)] resulted in triggering of >90% of combined chalky (MW + WB) phenotypes (Table�1). Endospermal starch accumulation of superior caryopses, which were used in all experiments in this study, occurs during 5–20 DAF (Ishimaru et�al. 2003). Temporal exposure to high temperature at 5 d intervals was conducted during 5–10 DAF, 10–15 DAF and 15–20 DAF as the early, middle and late storage phase, respectively. Nearly half of the grains had the combined chalky phenotype when exposed during the early storage phase, while a much lower frequency was noted in the exposure during middle and late storage phases (Table�1). The frequency of the BW type of chalk increased on exposure to high temperature at the later storage phase (Table�1). The white-belly type of chalky grains was at low frequency at any time of exposure (Table�1).

**Table 1:** Effect of temporal high-temperature stress on the frequency of the type of chalky grains

Timing of high-temperature treatment	Frequency of type of grains (%)						No. of grains used
	Perfect	Combined chalk (MW + WB)	MW	WB	Basal-white	White-belly	
3–35 DAF	0.0	90.2	8.2	0.0	1.6	0.0	61
5–10 DAF (early)	4.9	48.2	37.0	5.0	4.9	0.0	81
10–15 DAF (middle)	34.8	10.1	27.0	0.0	25.8	2.3	89
15–20 DAF (late)	21.7	11.3	11.3	9.3	40.2	6.2	97

### Amyloplast development in the central zone of starchy endosperm during the early storage phase

Amyloplast development appeared similar, with various sizes of single and compound amyloplasts, at 7 and 6 DAF in control and high-temperature conditions, respectively (Fig.�2A, C). While amyloplasts were fully packed without gaps between them at 9 DAF in control conditions (Fig.�2B), the starch granules were loosely packed with single and compound amyloplasts at 8 DAF in high-temperature conditions (Fig.�2D). These observations indicate that impaired amyloplast development at the CSE was determined by 8 DAF under the given high-temperature conditions.

**Fig. 2:**
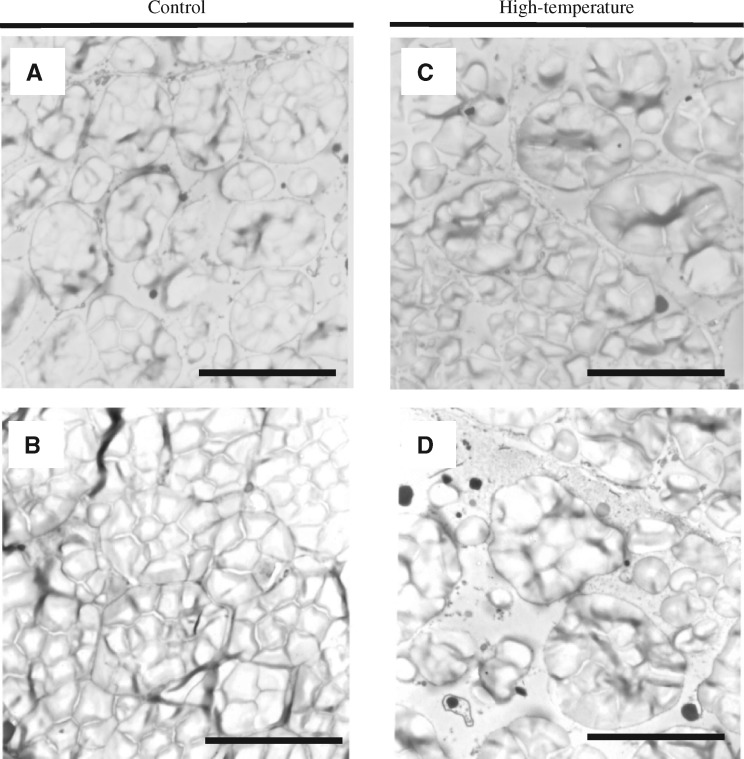
TEM observation of amyloplast development at the CSE zone in control and high-temperature conditions. 7 DAF (A) and 9 DAF (B) of control-treated grains. 6 DAF (C) and 8 DAF (D) of high-temperature-treated grains. Scale bars = 10 μm.

### LM for isolating endosperm tissues

Temporal exposure to heat stress during the storage phase (Table�1) clarified that the early storage phase (5–10 DAF) is the most sensitive in terms of chalky apprearance. To analyze the tissue-specific responses of expression of genes in key developing endosperm zones to heat stress, AL, CSE, LSE and DSE zones were microdissected from developing caryopses at 7 and 6 DAF in control and high-temperature conditions, respectively (Fig.�3A–C). Note that CSE and DSE zones correspond to the MW and WB type of chalk, respectively, in high-temperature conditions.

**Fig. 3:**
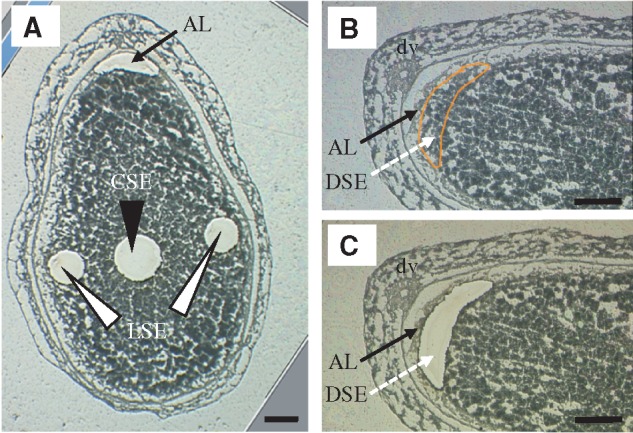
Isolation of fine endosperm tissues at AL, CSE and LSE (A), and DSE (B, C) by laser-microdissection. dv, dorsal vascular bundle. Scale bars = 200 μm.

### Gene regulatory networks and pathway analysis

After RNA extraction from dissected tissues based on Ishimaru et�al. (2007), a microarray experiment was conducted following the method of Takehisa et�al. (2012). Altogether, we identified a set of 563 differentially expressed genes between control and high-temperature conditions (Supplementary Table S2). Normalized expression values from control and heat-stressed microdissected tissues targeting AL and different zones of starchy endosperm such as the DSE, CSE and LSE, were considered to construct gene regulatory networks. The co-expression network was constructed using the WGCNA (weighted gene correlation network analysis) package in R software (Langfelder and Horvath 2008), which is a guilt-by-association approach. By applying the steps described in the Materials and Methods, signed network adjacency matrices were obtained by raising the Pearson correlation matrices to a power β = 12 which approximates scale-free topology. Then, the dissimilarity of the topological overlap matrix (TOM) was calculated based on the adjacent coefficient. Using this TOM calculation, average linkage hierarchical clustering was carried out using the flashClust R package shown as trees (dendrograms) in Fig.�4A, where each leaf (vertical lines) corresponds to a specific gene. For further analysis, a dynamic branch cutting method called ‘hybrid’ was used to cut the tree to generate modules (clusters). Modules on the bottom of the leaf are illustrated with different colors and represent the branches of the clustering tree (Fig.�4A). We identified five distinct modules with varying gene frequency as M1 (turquoise, size-178 nodes), M2 (brown, size-106 nodes), M3 (blue, size-149 nodes), M4 (green, size-47 nodes) and M5 (yellow, size-83 nodes). Heat maps of changes in gene expression in each module are indicated as color bands shown in Fig.�4A and Supplementary Fig. S2. The genes co-expressed at the M1 module were highly up-regulated under heat stress in AL and DSE, whereas the M2 module was up-regulated under heat stress in all tissue fractions. The M3 module represented genes which were down-regulated in heat stress in AL. The modules M4 and M5 were mostly down-regulated by heat stress across all tissues. The gene network structure of each co-expressed module is shown in Fig.�4B(a–e) and the annotation based on various databases is shown in Supplementary Table S2. Module M1 consisted of 178 genes (nodes) which fall into 14 categories of the Gene Ontology (GO) database highlighted in different colors (Fig.�4Ba). Three small hsp genes of 17.9, 17.7 and 22.7 kDa were found in the heat stress category. The neighboring genes of the hsp genes interacted abundantly with unknown genes, and cell- (organization, cell cycle, cell division), protein synthesis- and signaling- (calcium kinases, sugar) related pathway genes. A few interacting genes related to chromatin, galactose metabolism, lipid metabolism, etc. were found. The module M2 consisted of 106 nodes grouped into 14 categories shown in Fig.�4Bb. Altogether, 23 hsp-related genes were found of various types such as 16.9 kDa class I, 17.5 kDa class II, 17.9 kDa class III, hsp20, Oshsp17.3, hspLMW, hsp26, hsp70 and hsp (molecular chaperone). They also abundantly interacted with unknown, cell- and protein synthesis-related genes. A few interacting genes related to ethylene hormone synthesis, carbohydrate metabolism, lipid transfer protein (seed storage), signaling G-proteins, etc. have also been found. The module M3 had a total of 149 genes which fall into 16 categories (Fig.�4Bc). Both biotic and abiotic stress-related genes were found, which included hsp82, hsp101, hsp unspecified, and biotic mainly chitinase and reticulon. After the unknown category, the other abundantly interacting genes were in protein, glucose metabolism, transport and misc. catogeries. Module M4 consisted of a total of 47 nodes falling into 10 categories (Fig.�4Bd) out of which stress-related genes were hsp (lumen binding), hsp (GRP94) and biotic stress PR proteins. Apart from the unknown category, they interact with genes encoding transcription factors (SET-domain, C2H2, MAF-1), amino acid synthesis- (GABA, branched chain groups), redox- (dismutases, glutaredoxins, PDI) and transport- [NDP-sugars at the endoplasmic reticulum (ER)] related genes. Module M5 consisted of 83 genes out of which the stress related was only two hsp29 protein. Total genes fall into 10 categories, of which the abundance of unknown genes is 64%. The neighboring category genes interacting with the hsp29 gene were transport, development and misc. mostly (Fig.�4Be).

**Fig. 4:**
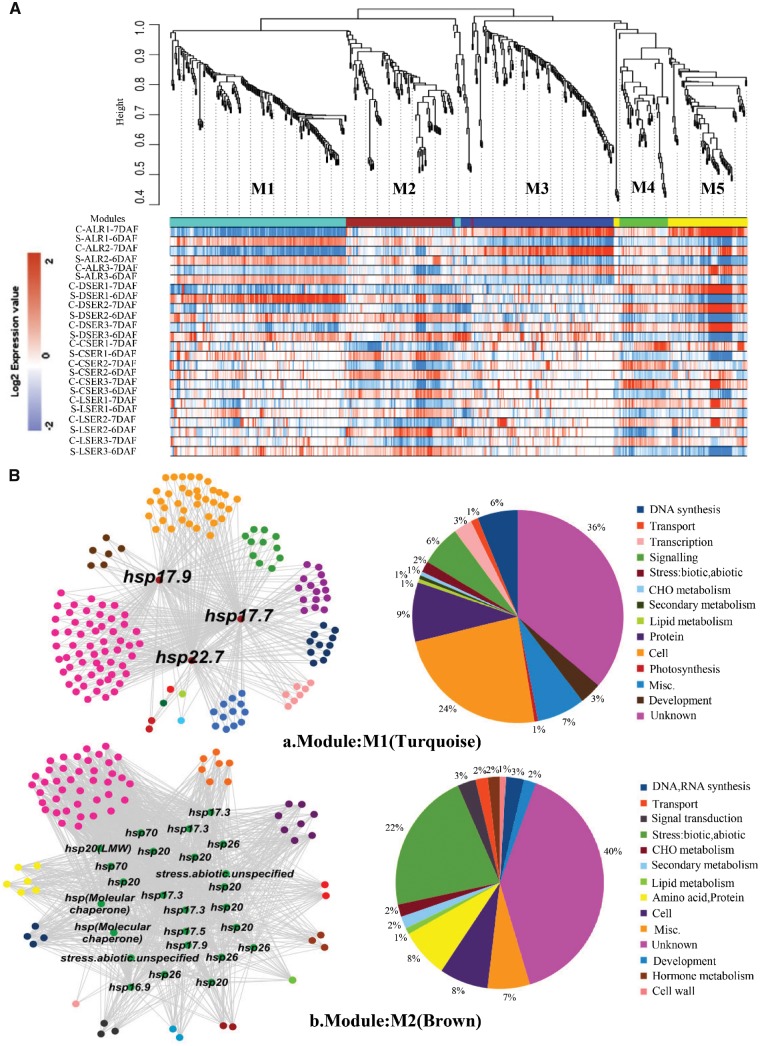

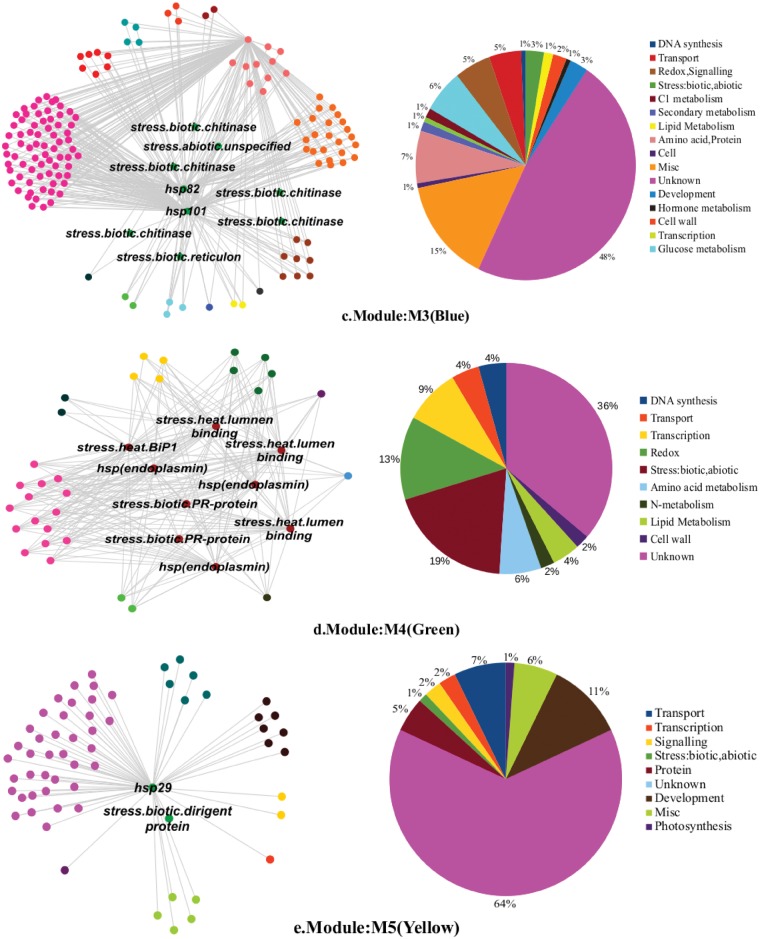
Gene dendrogram and corresponding heatmap (A) and gene regulatory network (B) of five distinct co-expressed modules: M1-turquoise, M2-brown, M3-blue, M4-green and M5-yellow; C-control, S-stressed and R1–R3-replicates, derived from differentially expressed genes across AL, DSE, CSE and LSE tissues under the control and high-temperature conditions. In a gene regulatory network (B), genes are represented by circular nodes and interaction by edges. Nodes are labeled as per the annotated functions which are sharing the same degree of connectivity, with varing strength of edges.

The log fold change of co-expressed module genes has been mapped on the overall metabolic and stress-related pathway of MapMan shown in Fig.�5A and B with an expression range of –2 (blue, down), 0 (white, neutral) to +2 (red, high). A contrasting difference in the expression pattern of stress-related genes along with other genes has been observed. Fig.�5A (a, b) shows that all the stress-related genes are up-regulated in both the pathways, while in Fig.�5B (c, d, e), the pattern is opposite. The log fold change and *P*-value of all mapped genes are shown in Supplementary Table S2.

**Fig. 5:**
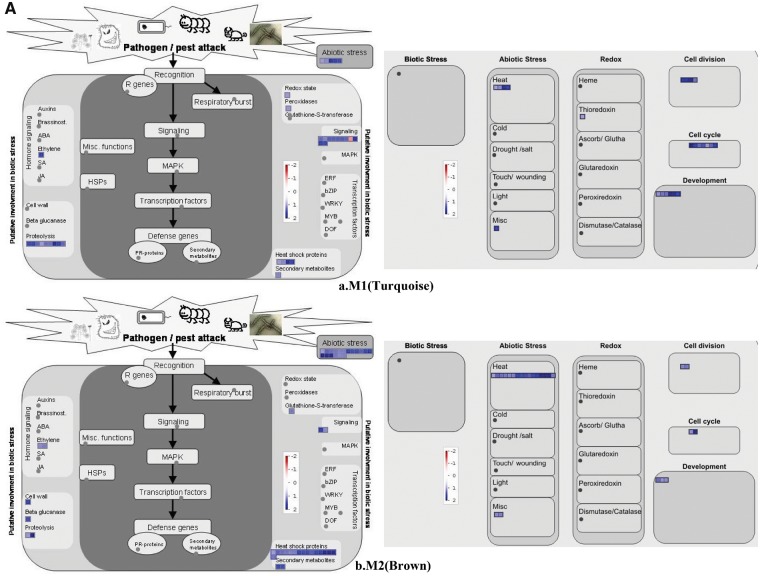

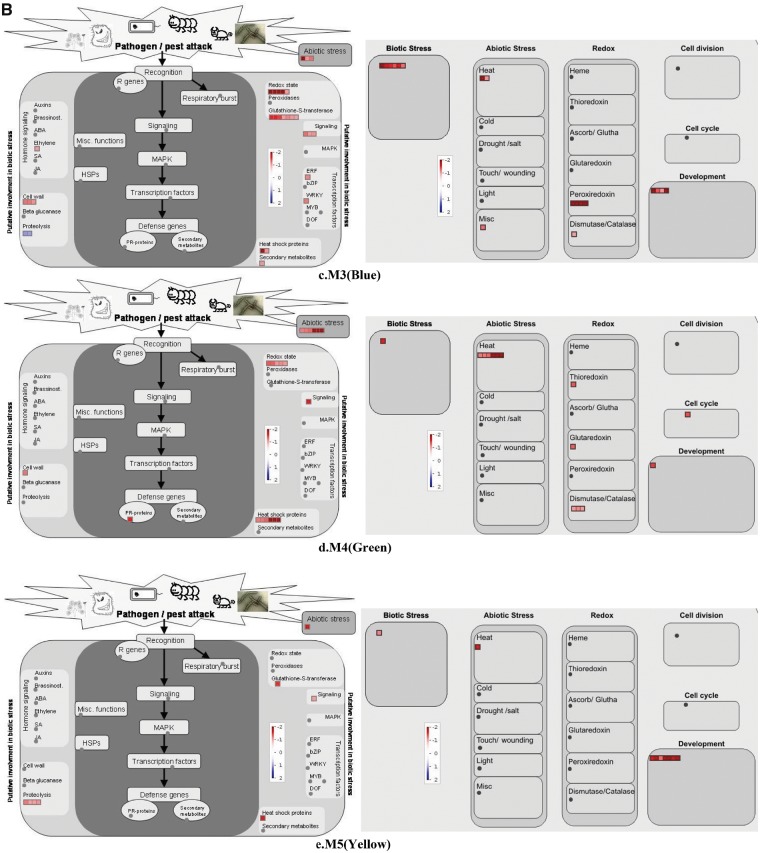
Mapman pathway overview of cellular response and stress pathway of modules up-regulated under heat stress (M1 and M2) and modules down-regulated under heat stress (M3–M5). logFC + 2 blue, up-regulated under stress treatment; logFC – 2 red, down-regulated under stress treatment.

### Reinforcing the importance of heat shock proteins that preferentially changed in CSE being linked with the MW type of chalk

The weighted gene co-expression analysis revealed that expression of a number of HSPs (molecular chaperones) was changed by heat stress. The M1 module contains the genes up-regulated in DSE, where the zone corresponds to the WB type of chalk. M3 represents genes which are down-regulated in AL, where the tissue is spatially far from the CSE zone. No particular module that contained the number of genes changed in the CSE zone was identified (Fig.�4A). There is, however, still a possibility to identify the key genes that preferentially changed in CSE, where the zone corresponds to the MW type of chalk (Fig.�1E). HSPs among endosperm tissues were selected from M2 and M4 modules, whose expression was up-regulated and down-regulated in all tissue fractions (Fig.�6). M2 contained several small HSPs, but their expression was also relatively up-regulated in other endosperm tissues under heat stress (Fig.�6A). In M4, expression of the endosperm lumenal binding protein gene (*BiP1*, classified as HSP70, AK119653) and endoplasmin (classified as HSP90, AK102478) was preferentially down-regulated in CSE under heat stress (Fig.�6B). This tendency was also true for *OsPDIL2-3* (AK062254), an isogene of PDI (Fig.�6B). Very interestingly, both *BiP1* and *OsPDIL2-3* are reported to be localized in the ER in rice grain (Muench et�al. 1997, Onda et�al. 2011). Endoplasmin is reported also to be localized in the ER in Arabidopsis leaves (Klein et�al. 2006). HSPs preferentially down-regulated in CSE seem to be ER-localized molecular chaperones.

**Fig. 6:**
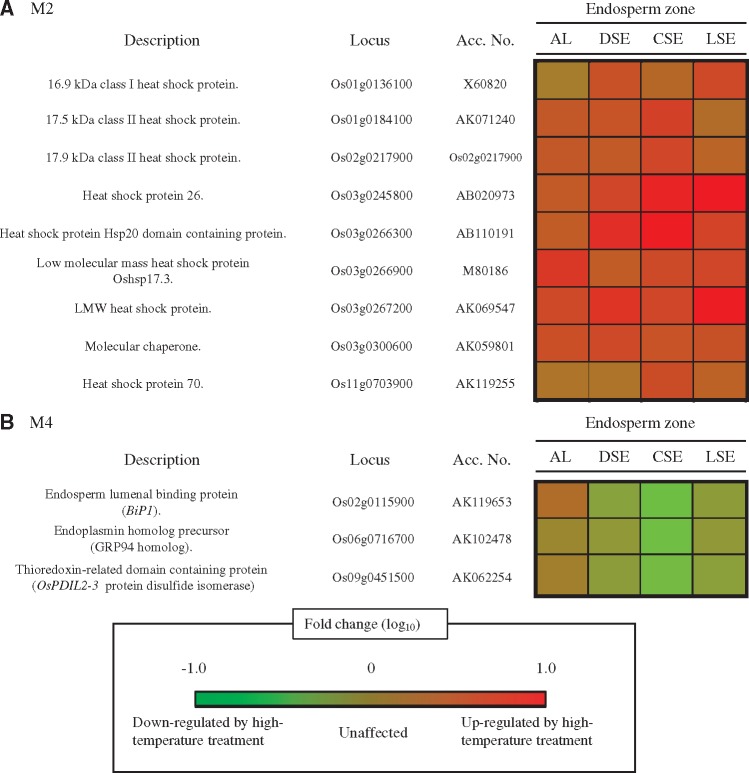
Spatial expression patterns of heat shock proteins in M2 and M4 among fine tissues of developing endosperm at the early storage phase.

### Morphological changes in aleurone cells under high-temperature conditions

Aleurone cells were examined using perfect grains from control conditions, and chalky grains with and without the WB type from high-temperature conditions (Table�2). The averaged cell number at the dorsal side was 3.53 in control conditions, while it was 5.33 in the WB type of grains in high-temperature conditions, a significant difference. Layer thickness and cell size in the dorsal side were also significantly greater in WB grain in high-temperature conditions. Notably, averaged cell number, layer thickness and cell size at the dorsal side were similar between perfect grains from control condition and grains without the WB type of chalk from high-temperature conditions (Table�2). Cell number at the lateral and ventral side was not different among grain types. These results clearly indicate that the WB type of chalk occurs concomitantly with the morphological change in dorsal aleurone cells.

**Table 2:** Cell number, layer thickness and cell size of the AL in the matured grains

Treatment	Grain type	Cell number			Layer thickness (�m)	Cell size (�m per cell)
		Dorsal	Lateral	Ventral	Dorsal	Dorsal
Control	Perfect	3.53b	1.09	1.00	76.9b	22.0b
High temperature	WB	5.33a	1.14ns	1.15ns	165.7a	31.3a
High temperature	Without WB	3.58b	1.00	1.00	82.6b	23.0b

Different letters indicate significance at the 5% level by Tukey’s test. ns, not significant.

### Changes in amylose content and prolamin molecules at the CSE zone

In the entire grain, amylose content was 17.1% and 16.3% in control and high-temperature conditions, respectively (Table�3). At the CSE zone, amylose content was significantly lower in high-temperature conditions (16.1%) than in control conditions (19.8%), implying that the lower activity of amylose synthesis might be involved in chalk formation at the CSE zone. The different molecular sizes (10, 13 and 16 kDa) of prolamin were also investigated from entire grains and the CSE zone from control and high-temperature conditions (Fig.�7A). Values are shown as the ratio of high temperature to control, which was calculated from the signal intensity of immunologically detected prolamin polypeptides (Fig.�7B). In the entire grain, the ratio of 10 and 13 kDa prolamin content greatly decreased, while that of 16 kDa was not changed (Fig.�7C). The ratio of 16 kDa prolamin was similar between the entire grain and CSE (0.87), while those of 10 and 13 kDa were reduced to 0.68 and 0.37 at the CSE zone, respectively (Fig.�7C). Among different molecular sizes of prolamin, 13 kDa prolamin was most sensitive to heat stress, followed by 10 kDa prolamin.

**Table 3:** Amylose content (%) of entire grains and the central starchy endosperm between control and high-temperature conditions

	Entire grain	CSE
Control	17.1ns	19.8^a^
High temperature	16.3	16.1

^a^ Significant between conditions at the 0.1% level by *t*-test. ns, not significant.

Amylose content is adjusted to a 15% moisture basis.

**Fig. 7:**
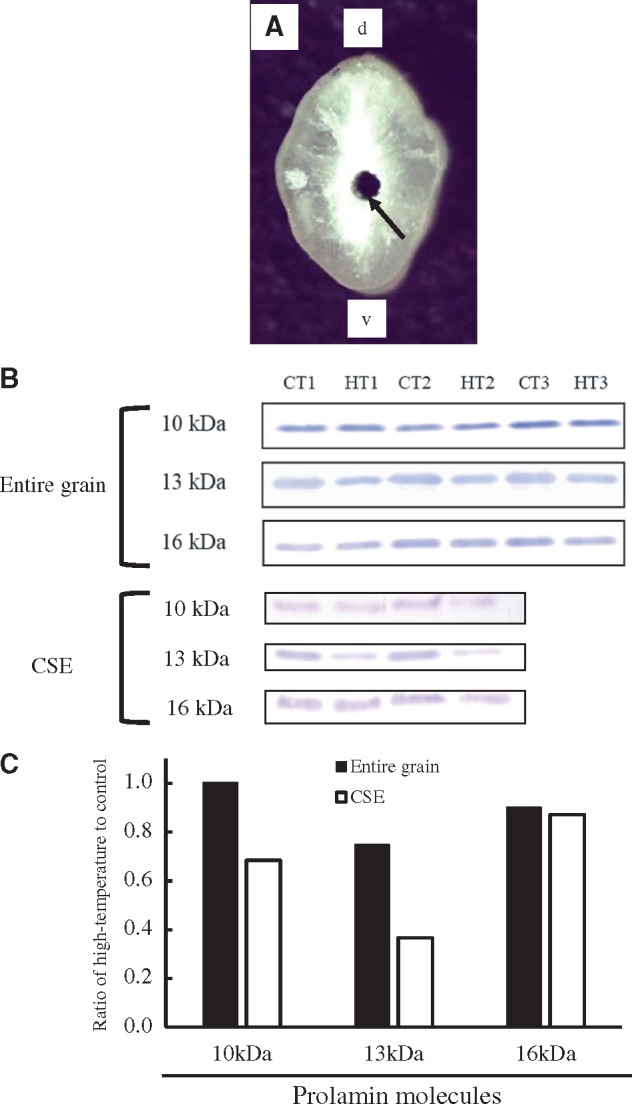
Differences in prolamin molecules in perfect grains from the control condition and MW + WB (or MW phenotype only) grains from the high-temperature condition. (A) Transverse section after isolation of the CSE zone of high temperature-treated grain by a fine-tipped drill. The translucent CSE zone of the perfect grains is also isolated. Scale bar = 10 mm. (B) Detection of prolamin polypeptides by Western blot. CT, control temperature; HT, high temperature. Each number indicates the biological replication. (C) Quantification of the molecular weights of prolamin of entire grains and the CSE zone. Values are indicated as the ratio of high-temperature to control treatment.

### Characterization of chalky grains in *BiP1*-suppressed lines

Our microarray analysis showed the down-regulation of *BiP1* preferentially in the CSE zone under heat stress (Fig.�6B). Transgenic evidence showed that *BiP1* affected prolamin formation (Yasuda et�al. 2009, Wakasa et�al. 2011). We further attempted to validate the effect of *BiP1* on chalk formation with artificial *BiP1*-suppressed lines generated by following the methods of Hakata et�al. (2012). Suppression of *BiP1* mRNA in RNA interference (RNAi) lines ranged from 8.2% to 32.8% compared with the vector control (Fig.�8C). All lines produced opaque phenotype grains at high frequency (Fig.�8A) with a large decrease in grain dry weight (Supplementary Table S3). Note that the opaque phenotype of grains was inherited from KD9 (T_0_ plants) to KD9-1 (T_1_ plants). Opaque phenotype grains had a reduced amylose content (Supplementary Table S3). SEM observation revealed the existence of various sizes and shapes of single and compound irregular amyloplasts in *BiP1*-suppressed lines (Fig.�8B). Quantitative reverse transcription-PCR (qRT-PCR) analysis of starch biosysnthesis genes showed that their expression level was not always down-regulated in developing grains of *BiP1*-suppressed lines (Fig.�8C). The results indicated the critical effect of *BiP1* on grain chalk formation, accompanying some similar grain characteristics in the MW type of chalk.

**Fig. 8:**
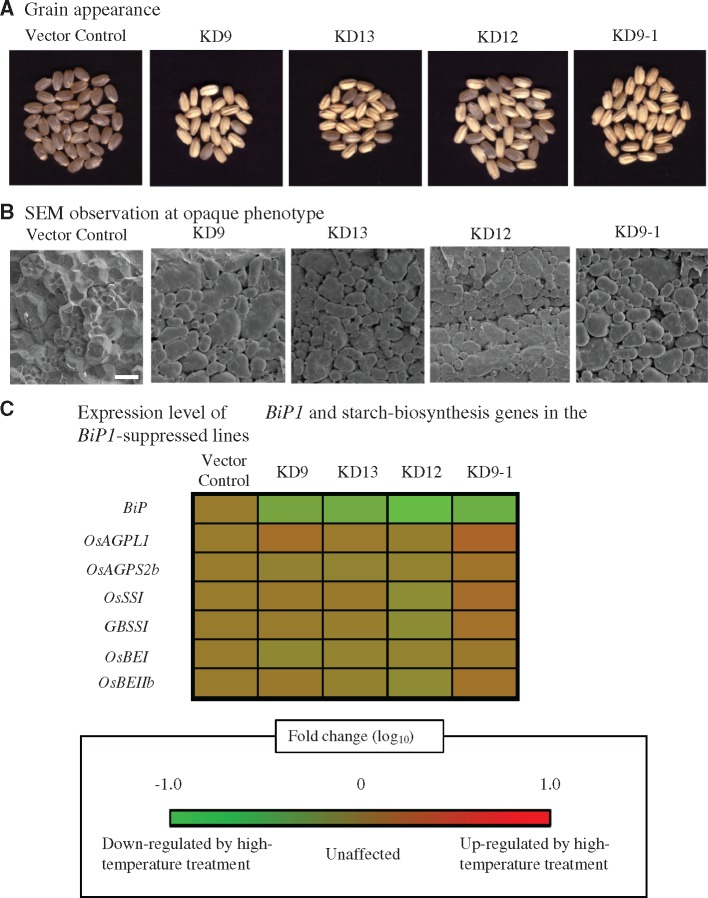
Grain appearance (A), amyloplast at the CSE zone in matured grains (B) (scale bar =10 μm) and qRT-PCR analysis of developing grains at the early storage phase in *BiP1*-suppressed lines (C). In (C), the epression level in the vector control was set at 0 in each gene, and the expression level of each *BiP1*-suppressed line was compared with that of the vector control.

## Discussion

### Pathways affecting starch biosynthesis and degradation, and storage protein synthesis at the early storage phase under heat stress

Loosely packed single and compound amyloplasts cause a chalky appearance in common with MW and WB grains (Supplementary Fig. S1B, C; Tashiro and Wardlaw 1991). The early storage phase (5–10 DAF) was the most sensitive time for both MW and WB types of chalky grains under the given high-temperature conditions (Table�1). Amyloplast development initiates at 4–5 DAF in the entire starch endosperm, proceeds to the active packing of starch granules until 10 DAF at the CSE zone, and then spreads to the outer layer of starchy endosperm such as the LSE and DSE zones (Hoshikawa 1968). Distinct chalk phenotypes in MW and WB grains start to be formed through impaired development of amyloplasts. DSE and CSE zones at the early storage phase were determined as key fine tissues to understand the molecular mechanisms for the formation of WB and MW types of chalk, respectively.

Previous studies on rice grain development under heat stress have identified the heat-responsive genes related to starch biosynthesis and degradation, and storage protein synthesis (Lin et�al. 2005, Yamakawa et�al. 2007, Liao et�al. 2014) using the RNA/protein extracted from the entire grains. Since impaired amyloplast development causes a chalky apperance, it was assumed that the number of genes for starch biosynthesis and degradation would be enriched in the differentially expressed gene sets among the five modules. The LM-based expression analysis, however, indicated that only sucrose synthase (*SUS1*; Hirose et�al. 2008) was listed as a heat-responsive (up-regulated) gene in M2 (Supplementary Table S2). This result suggests that changes in the expression level of genes for starch biosynthesis and degradation is minimal to form MW and WB types of chalk at the early storage phase. With regards to the genes for storage protein synthesis, many genes for prolamin and glutelin were down-regulated by heat stress in M5 (Fig.�4Be; Supplementary Table S2). The results for storage protein synthesis were basically consistent with previous studies (Yamakawa et�al. 2007, Liao et�al. 2014), suggesting that the expression of genes for prolamin and glutelin is highly responsive to heat stress in any zone of young developing endosperm. The present LM-based tissue-specific expression analysis documents the different responses of genes for storage compounds to heat stress at the early storage phase.

### Possible molecular mechanism in forming the WB type of chalk by heat stress

High temperature stress accelerates the dry matter accumulation in the developing grains (Yamakawa et�al. 2007, Ishimaru et�al. 2009). A greater amount of assimilates for storage compounds (i.e. starch, storage proteins and lipids) is assumed to be loaded in young developing endosperm under heat stress. Dorsal aleurone cells (AL) are adjacent to the terminus of the dorsal vascular bundle, thereby AL functions as the main route of assimilate supply to developing endosperm (Oparka and Gates 1981). Dorsal starch endosperm (DSE), where the WB type of chalk is formed (Fig.�1F), is spatially connected with AL, but the function and storage products are quite different from each other (Ishimaru et�al. 2003). Our LM-based expression analysis showed that the genes highly up-regulated in AL and DSE were classified into M1 (Fig.�4A), which consisted of diverse biological processes such as regulation of the cell cycle and organelle organization (Figs.�4Ba, 5). These results from expression analyses suggest that the WB type of chalk is formed concomitantly with the morphological changes in adjacent AL tissues. Our morphological study revealed that the increased cell layer and sizes in AL were closely associated with the WB type of chalk (Table�2). We noticed that preferential up-regulation of cellularization pathways in AL under heat stress might be under the influence of small HSPs (Fig.�5A). In agreement with this, previous reports highlighted that small HSPs were preferentially up-regulated relating to the increase in chalky phenotypes (Lin et�al. 2005). Furthermore, M3 contains the genes of stress responsive pathway and high molecular weight (HMW) HSPs down-regulated by heat stress in AL. Aleurone cells start to differentiate at 5 DAF and continue thereafter (Ishimaru et�al. 2003). Down-regulation of stress response pathway and HMW HSPs might add to the vulnerability of the AL zone to the heat stress at the early storage phase, resulting in the drastic morphological changes in cell number and cell size. This study sheds new light on the possible involvement of dorsal aleurone cells in the formation of the WB type of chalk through changes in expression of genes in M1 and M3. A causal relationship of changes between AL and DSE in the WB type of chalk needs to be further investigated.

There is a clear genetic variation in WB grains among Japonica group varieties (Kobayashi et�al. 2007, Tabata et�al. 2007). Forward genetics have narrowed down the quantitative trait loci (QTLs) for WB grains to a 1.9 Mbp region on chromosome 6 (Shirasawa et�al. 2013). Three genes in M1, i.e. protein of unknown function DUF581 family protein (AK102200), early nodulin 93 ENOD93 protein family protein (AB018376) and hypothetical protein (AK061597), are localized in this QTL region (Fig.�4Ba; Supplemenatry Table S2). In contrast, no gene listed in M3 is localized in the QTL region of Shirasawa et�al. (2013). The effect of these candidate genes in M1 on the WB type of chalk under heat stress remains to be elucidated.

### ER-localized HSPs could be one of the key regulatory networks for the formation of the MW type of chalk under heat stress

Transmission electron microscopy (TEM) observation provided clear evidence that the MW type of chalk is formed in the CSE zone during the early storage phase (Fig.�2). Our previous study using magnetic resonance imaging (MRI) showed the lower water distribution in the CSE zone of young developing grains grown under heat stress (Ishimaru et�al. 2009). Since a change in water distibution is spatially and temporally associated with starch accumulation (Horigane et�al. 2001), the rapid decline of water may suggest water stress at the CSE zone. Detailed expression pattern analysis found the preferential down-regulation of three HMW HSPs (*BiP1*, endoplasmin and *OsPDIL2-3*) in the CSE zone (Fig.�6B). BiP and PDI are the ER-localized molecular chaperones and have critical roles in assisting protein folding and maturation, and thus BiP and PDI are essential in quality control in the early secretory pathway in plant species (Liu and Howell 2010). BiP and PDI are also essential for forming the seed storage proteins in rice endosperm. Prolamin is stored in type-I protein bodies (PB-Is), whereas glutelin is stored in type-II protein bodies (PB-IIs) (Tanaka et�al. 1980). Notably, *BiP1* and *OsPDIL2-3* localize in the ER-derived PB-I, having a critical role in prolamin formation (Yasuda et�al. 2009, Onda et�al. 2011). To validate the effect of *BiP1* on seed phenotype, knockdown (KD) lines of *BiP1* were generated using the endosperm-specific promoter. All KD lines produced opaque grains with single and irregularly compounded amyloplasts (Fig.�8A, B) as reported by Wakasa et�al. (2011), and this study clarified that aberrant amyloplast development and significant reduction in amylose content (Supplementary Table S3) did not result in the great suppression of genes related to starch biosynthesis (Fig.�8C). Prolamins of 10 and 13 kDa were reduced in the CSE zone under heat stress (Fig.�7), supporting the evidence of reduced expression of *BiP1* in the CSE zone due to heat stress. As a consequence, it was validated that significant reduction in *BiP1* mRNA during seed development resulted in aberrant amyloplast development, and a reduction in amylose and prolamin molecules using heat-induced MW grains and *BiP1*-suppressed transgenic lines. We indicate the possibility that reduced expression of *BiP1* increased the unfolded and immature proteins, which are important for starch and prolamin synthesis, thereby causing the MW type of chalk in the early storage phase under heat stress. Effects of endoplasmin and *OsPDIL2-3* on the grain phenotype need to be examined by developing KD transgenic plants. Reduced expression of *BiP1* implies that ER-localized HSPs could be one of the key regulatory networks to form the MW type of chalk under heat stress during the early storage phase, associated with altered redox, nitrogen and amino acid metabolism (Fig.�5Bd). The relationship between possible water stress (Ishimaru et�al. 2009) and down-regulation of ER-localized molecular chaperones in the CSE zone (Fig.�6B) is still a matter of debate. In the notched-belly mutant with the white-belly type of chalky grains, Lin et�al. (2014) reported the down-regulation of *BiP1* and *OsPDIL2-3* genes. The key regulatory network in forming the MW type of chalk may be similar to that in forming the white-belly type of chalk regardless of the spatial difference in the chalk zone in rice endosperm.

Hakata et�al. (2012) reported the critical role of some of the key genes for α-amylase in forming MW grains under heat stress. Expression of genes for α-amylase, however, did not occur in the CSE zone at the early storage phase (Ishimaru et�al. 2009). This discrepancy is probably because the expression of key α-amylase (*Amy1A*, *Amy3A*, *Amy3D* and *Amy3E*) genes is high after the middle storage phase under heat stress (Yamakawa et�al. 2007, Hakata et�al. 2012), when starch accumulation is completed at the CSE zone (Fig.�2B). α-Amylase is assumed to play a critical role in degrading the synthesized starch after the middle storage phase for the generation of a carbon source during ripening (Hakata et�al. 2012). At the early storage phase, endospermal starch accumulation is still active around the central zone (Fig.�2A;Ishimaru et�al. 2003), and our expression analysis at the early storage phase showed the suppressed expression of genes for ER-localized molecular chaperones (*BiP1*, endoplasmin and *OsPDIL2-3*) under heat stress (Fig.�6B). In terms of chalk formation at the CSE zone under heat stress, we hypothesize that ER-localized HSPs are the key element to suppress the initial amyloplast development evidenced by a transgenic approach (Fig.�8A, B) and, once starch accumulation is completed in the central zone, starch is degraded by α-amylase probably for survival through the adaptation under heat stress (Hakata et�al. 2012). Complex molecular physiological mechanisms in forming the MW type of chalk under heat stress are documented from the present study. Alteration of ER-localized HSPs under heat stress may impair amyloplast development and packaging of the storage proteins prolamins involved in chalk formation in the early to middle seed storage phase. Activation of α-amylase with suppression of the starch biosynthetic pathway during the middle to late seed storage phase affects susequent chalk formation.

### Conclusion

In the present study, integrated experiments on morphology, comprehensive expression analyses and measurements of storage compounds were carried out focusing on the MW and WB types of chalk formed at the early storage phase under heat stress. Although impaired amyloplast development causes a chalky appearance, the changes in expression of genes for starch biosynthesis and degradation were minimal. Through microdissection-based transcriptome analysis, we propose the molecular physiological hypothesis of causing MW and WB types of rice chalky grains under heat stress (Fig.�9). The important gene regulatory network related to the stress response pathway under heat stress was found to be upstream biological regulators rather than starch biosynthesis and degradation genes themselves. The WB type of chalk is formed concomitantly with the down-regulation of genes for the stress response pathway at AL and with the up-regulation of genes for cell division and expansion at AL and DSE. As regards the MW type of chalk, expression of ER-localized molecular chaperones was down-regulated in the CSE zone and evidence that *BiP1* plays a critical role in regulating both amyloplast development and prolamin synthesis under heat stress was provided using KD plants having similar grain characterics to the MW type of chalk. The overall outcome highlights the different molecular physiological mechanisms underlying MW and WB types of chalk at the early storage phase.

**Fig. 9:**
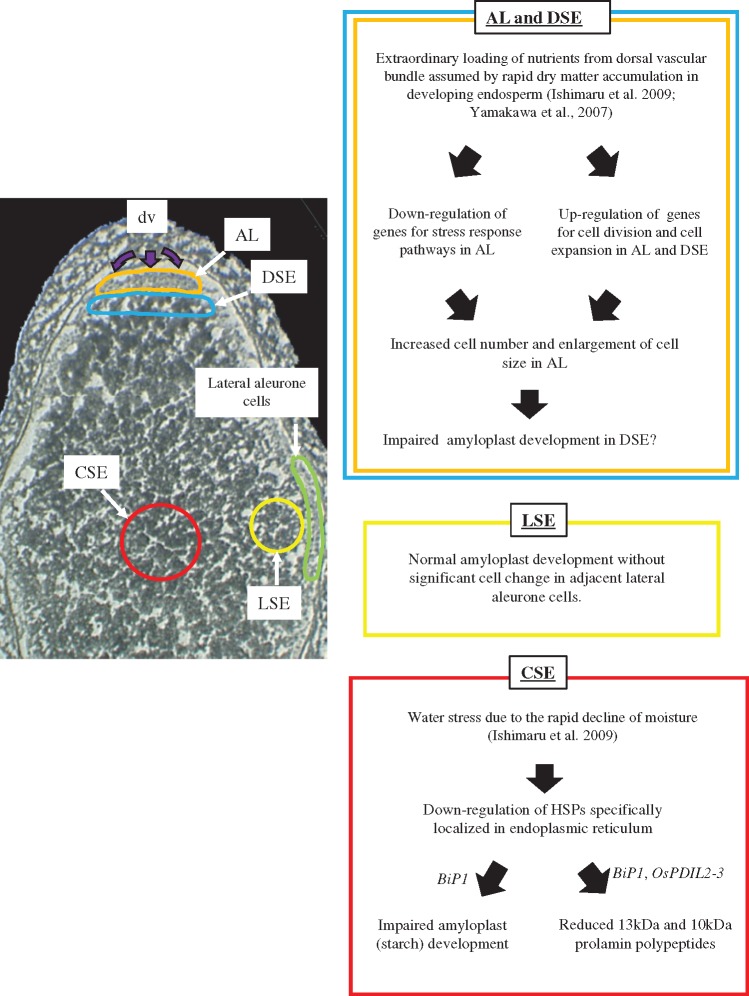
A hypothesis of what causes causing MW and WB types of chalk at the early storage phase from molecular physiological aspects. Purple arrows indicate the loading of nutrients from the dorsal vascular bundle (dv) to young developing endosperm.

## Materials and Methods

### Plant materials

A Japonica group rice cultivar, ‘Koshihikari’ (*Oryza sativa* L.), was used to test the impact of high temperature during seed development. A 4-week-old seedling was transplanted into 0.02 m^2^ pots with fertilizer application of 0.5–2.3–2.2 g of N–P_2_O_5_–K_2_O as basal dressing. Plants were grown outdoors until flowering. Top dressing (0.4 g of nitrogen per pot) and chemical spraying were applied as necessary. Flowering spikelets were marked with fine-tipped pens. Developing caryopses and matured grains from the first to the fourth primary rachis branches counted from the top of the panicle, which are called ‘superior caryopses/grains’ (Ishimaru et�al. 2003), were used in this study. Two to three large panicles were selected per pot.

### Temperature treatment

At 3 DAF, pots were moved to the naturally illuminated temperature-controlled chamber. Control and high temperature conditions were set at 26/20�C and 33/27�C (day/night), respectively. Day and night lengths were 13 h (06.00–19.00 h) and 11 h (19.00–06.00 h), respectively. Plants were kept in the chambers until 30–35 DAF, and marked spikelets were harvested. Temporal exposure to heat treatment for 5 d was also carried out with a 5 d interval starting from 5 DAF until 20 DAF; marked spikelets were exposed to high temperature during either 5–10, 10–15 or 15–20 DAF. Each temporal treatment had three plants. After the treatment, pots were returned to the control conditions until harvest.

### Evaluation of grain apprearance

The appearance of matured grains was visually evaluated and grains were classified into perfect, MW (synonymous to the white-core type), WB, BW and white-belly types of chalk according to the criterion of Tashiro and Wardlaw (1991). Note that combined types of chalky grain (MW + WB) were counted into a different category from those of the sole types of chalk.

### Laser-microdissection

Developing caryopses was fixed with an ice-cold mixture of ethanol–acetic acid (3:1) and embeded in 2% carboxymethylcellulose following the method of Ishimaru et�al. (2007). Transversal sections (8 �m thickness) were made at the median part of developing caryopses using a cryo-microtome (CM1850, Leica). AL, DSE, CSE and LSE at 7 DAF from control and at 6 DAF from heat-stressed tissues were dissected using an AS LMD system (Leica Microsystems) by considering the accelerated growth rate of caryopses in high-temperature conditions (Ishimaru et�al. 2009).

### Two-color microarray and data analyses

Extraction, integrity assessement and quantification of RNA were based on Ishimaru et�al. (2007). The microarray experiment was conducted based on the method of Takehisa et�al. (2012). Total RNA (5.0 ng) was amplified to obtain complementary RNA (cRNA) using the two-color Quick Amp Labeling kit (Agilent Technologies), according to the modified manufacturer’s instructions. RNA extracted from control and high-temperature conditions was labeled with cyanine-3 (Cy3)-CTP and cyanine-5 (Cy5)-CTP, respectively. Three biological replicates were prepared from high-temperature conditions as well as under control conditions. A 1250 ng aliquot of labeled cRNAs was fragmented and hydridized on a slide glass of the rice 4 � 44 K microarray RAP-DB (G2519F#1524; Agilent Technologies). Slides were scanned on an Agilent G2505B DNA microarray scanner, and the background of the Cy3 and Cy5 raw signals was corrected using the Feature Extraction (ver. 10.5.1.1, Agilent Technologies). The two-channel Agilent microarray expression data of 12 microdissected samples of AL, DSE, CSE and LSE at 7 DAF from control and at 6 DAF from heat-stressed tissues consisting of three replicates were further processed (ratio computation, log transformation and baseline transformation) using the software GeneSpring GX version 12 (Agilent). After quality check (QC) validation and data processing, a total of 24 normalized signal value samples with 42,537 probes were selected for further analysis (Supplementary Table S1). The differentially expressed genes were analyzed between the control and heat-stressed samples from individual tissue fractions. The Limma R (Ritchie et�al. 2015) package is used following the empirical Bayes method (Smyth 2004) to shrink the probe-wise sample variances towards a common value and to augment the degrees of freedom for the individual variances. The *P*-value adjustment method was used (Bonferroni 1936). The top-ranked genes having an adjusted *P*-value ≤0.05 and log_2_ fold change above � 1 were selected.

### Gene regulatory network analysis and identification of enriched functional categories of genes regulated under heat stress from the key modules

The WGCNA (Langfelder and Horvath 2008) method was used to identify the clusters (modules) of densely connected correlated genes and for derivation of the co-expression networks describing the pairwise relationships (Pearson) among differentially expressed transcripts between control and heat stress. The correlation matrix (coefficient <0.75) was transformed into a matrix of connection strengths (an adjacency matrix) by raising the correlation matrix to the power β of 12, which was interpreted as a soft threshold of the correlation matrix. Following the TOM, the similarity algorithm for the signed network of the adjacency matrix is converted to the TOM. Genes were hierarchically clustered based on TOM similarity. Modules with <30 genes were merged into their closest larger neighbor module. The visualization of the co-expression network was created by using Cystoscope software (Shannon et�al. 2003). GO enrichment of various sets of genes represented in each module was performed using the BINGO tool (Maere et�al. 2005) with Arabidopsis as the reference gene model. The enrichment analyses were performed with a *P*-value of <0.05 after applying Bonferroni correction. The log_2_ fold changes between the genes of heat stress and control were visualized using MapMan software (Thimm et�al. 2004). The genes were mapped with the regulatory and cellular response pathways, and the fold of differential expression was visualized within the respective binds of the MapMan pathway chart (Supplementary Table S2).

### Amylose content

20.0 and 2.0 mg of rice powder were collected by milling the entire grains and by drilling the CSE zone of the grains, respectively. The apparent amylose content was determined by the iodine absorption method (Juliano 1971).

### Determination of prolamin molecules

Total protein was collected from the entire and the chalky central portion of matured grains. Entire matured grains were homogenized in SDS buffer containing 62.5 mM Tris–HCl (pH 6.8), 4 M urea and 2% (w/v) SDS supplemented with 0.1 M dithiothreitol (DTT). Powder from the CSE zone was also collected by a fine-tipped drill, then the collected powder was homogenized with SDS buffer. Three and two replicates were prepared for the analysis of entire matured grains and the CSE portion, respectively, from the control and heat-stressed grains. The subsequent methods for protein extraction, SDS–PAGE and Western blot were based on Saito et�al. (2009). The signals immunologically detected by each antibody were quantified with electrophoresis documentation and the EDAD 290 analysis system (Kodak).

### Microscopic observation

*Stereo microscopy.* Median transversal sections of matured grains from control (perfect translucent grains) and high-temperature conditions (MW + WB grains) were made with a sharp razor, viewed under a stereo microscope (SZX12, Olympus) and photographed with a digital camera (E-330, Olympus).

*Light microscopy.* A median transversal section (1 mm thickness) of matured grains was made from control (perfect translucent grains) and high-temperature conditions (WB + MW and without WB grains). Note that grains with a clear WB phenotype but a slight MW phenotype were selected from WB + MW grains for this observation. MW and BW grains with just one type of chalk were used as ‘without’ WB grains. Samples were fixed with FAA (formalin:acetic acid:50% ethanol = 1:1:18), dehydrated in a graded ethanol series and embeded in Paraplast Plus (McCormick Scientific). Transversal sections (10 �m thickness) were cut with a microtome (RM2145, Leica), deparaffinized in xylene and a graded ethanol series, and stained with 0.05% (w/v) toluidine blue-O. Specimens were viewed under a digital microscope (VB-7000, KEYENCE), and the number of aleurone cells and the thickness of aleurone layers were examined.

*Scanning electron microscopy.* Transversal sections of matured grain were attached to alminum specimen stubs, and the specimen’s cut surface was coated with gold using an ion sputtering device (JFC-1100E, JOEL) under vacuum. The translucent and chalky portions were observed with a scanning electron microscope (Real Surface View VE-7800, KEYENCE).

*Transmission electron microscopy.* The experimental procedure is based on the method of Saito et�al. (2009). Developing caryopses at 7 and 9 DAF from control conditions and at 6 and 8 DAF from high-temperature conditions were cut into small pieces. Samples were fixed twice with 4% (w/v) paraformaldehyde in 0.1 M sodium phosphate buffer (pH 7.2), dehydrated in a graded ethanol series and embeded in LR White resin (London Resion). Ultrathin sections were cut with a diamond knife using a Leica Ultracut UCT and mounted on nickel grids. Specimens were stained with 2% (w/v) uranyl acetate, and viewed under a transmission electron microscope (JEM-1220, JEM) at 100 kV.

### Development of *BiP1* knockdown transgenic plants

The endosperm-specific *BiP1* KD transgenic plants were generated using a Japonica group rice cultivar, ‘Nipponbare’ (*O. sativa* L.) based on the method of Hakata et�al. (2012). Briefly, the 254 bp 3'-untranslated region was amplified by PCR with *Bam*HI and *Xba*I–*Sac*I restriction sites at the respective ends using the full-length cDNA clone, AK119653 (provided by the National Institute of Agrobiological Sciences); the primer set is listed in Supplementary Table S4. The fragment was inserted into the corresponding restriction sites (between *Xba*I and *Bgl*II sites and between *Bam*HI and *Sac*I sites) of the pZH2B10ik binary vector harboring a prolamin promoter-driven RNAi cassette (Kuroda et�al. 2010). The RNAi vector and the empty vector, pZH2B (used as a control), were introduced into *Agrobacterium tumefaciens* strain EHA101 by electroporation, and then subjected to rice transformation (Toki 1997). Four T_0_ lines (vector control, KD9, KD13 and KD12) were generated, then two lines (vector control and KD9-1) were advanced to the T_1_ generation. A total of five lines (T_0_: KD9, KD13 and KD12; T1: vector control and KD9-1) with 4–6 plants per line were grown in a naturally illuminated closed chamber set at a temperature of 27/21�C (12 h each, day and night). Developing superior caryopses at the early storage phase were sampled for expression analysis. Total RNA was extracted from entire developing caryopses following the method of Chang et�al. (1993). The first-strand cDNA mixture was prepared using 5 �g of total RNA, and qRT-PCR was performed as described previously (Ishimaru et�al. 2009). The transcript level of each gene was normalized with that of 18S rRNA (Kim et�al. 2003). At maturity, translucent and chalky (opaque) grains were visually selected from the control and four *BiP1* knockdown lines, respectively. SEM observation and measurement of amylose content were condcuted as described above. Grain dry weight was measured after drying at 80�C for 3 d.

### Statistical analyses

Significant differences in the amylose contents between control and high temperature condition (Table 3) were analyzed by a *t*-test. Significance of the means for cell morphology in AL (Table 2) and grain dry weight and amylose content among *BiP1*-suppressed lines (Supplementary Table S3) was tested by analysis of variance (ANOVA) followed by Tukey–Kramer’s test using STATISTIX ver. 9.0 (Analytical Software).

## Supplementary Data

Supplementary data are available at PCP online. All microarray data can be accessed from the GSE 122115 NCBI GEO repository.

## Funding

This work was supported by the National Agriculture and Food Research Organization (NARO) [a Grant-in-Aid (Jitsuyo Idenshi, No.1204), Japan to T.I.] and the Bill & Melinda Gates Foundation [STRASA phase III to N.S.].

## Supplementary Material

Supplementary Figure S1

Supplementary Figure S2

Supplementary Figure S3

Supplementary Table S1

Supplementary Table S2

Supplementary Table S3

Supplementary Table S4
