# Supplementary material for: Laser Microdissection-Based Tissue-Specific Transcriptome Analysis Reveals a Novel Regulatory Network of Genes Involved in Heat-Induced Grain Chalk in Rice Endosperm
Source: Plant Cell Physiol. 2018 Dec 4;60(3):626–42. doi: 10.1093/pcp/pcy233 (PMC6400107; doi:10.1093/pcp/pcy233)
Supplement: Supplementary Figure S1 [file pcy233_supplementary_figure_s1.pdf]

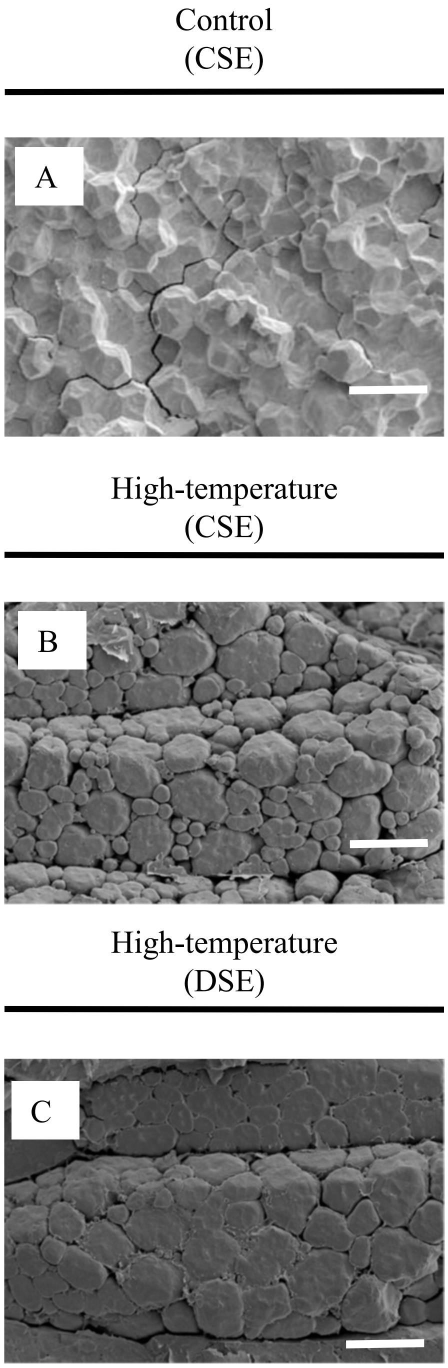

Supplementary Figure S1 SEM observation at CSE zone (A, B) and at DSE zone (C) of translucent and chalky part of matured grains from control and high-temperature condition. Note that packing of amyloplast at translucent DSE was identical to CSE in control condition. Bars = 20 µm.
