# Supplementary material for: Laser Microdissection-Based Tissue-Specific Transcriptome Analysis Reveals a Novel Regulatory Network of Genes Involved in Heat-Induced Grain Chalk in Rice Endosperm
Source: Plant Cell Physiol. 2018 Dec 4;60(3):626–42. doi: 10.1093/pcp/pcy233 (PMC6400107; doi:10.1093/pcp/pcy233)
Supplement: Supplementary Figure S2 [file pcy233_supplementary_figure_s2.pdf]

ISHIMARU ET AL. SUPPLEMENTARY FIGURE S2

A.Upregulated at heat stress

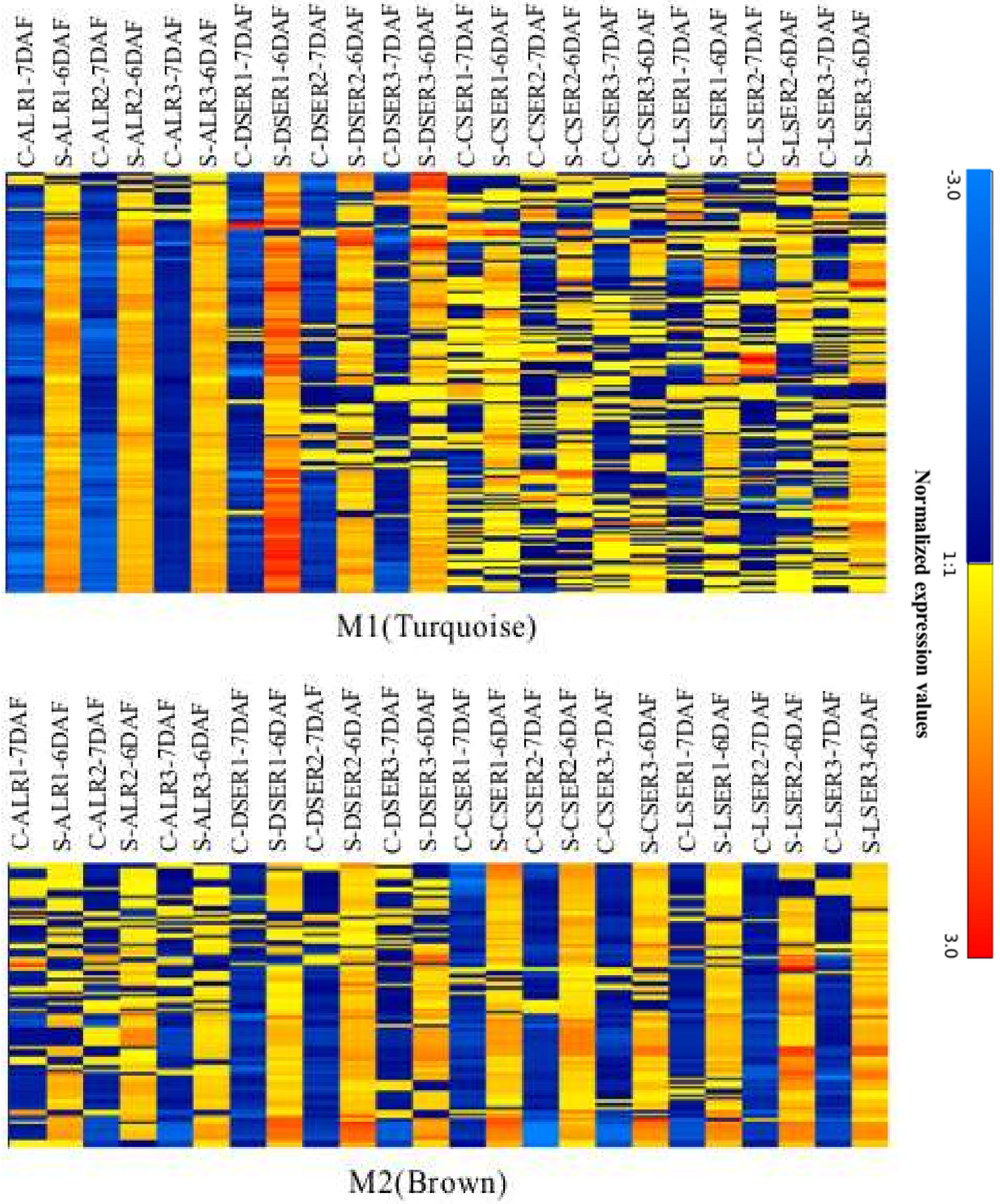

ISHIMARU ET AL. SUPPLEMENTARY FIGURE S2 (CONTINUED)

B.Downregulated at heat stress

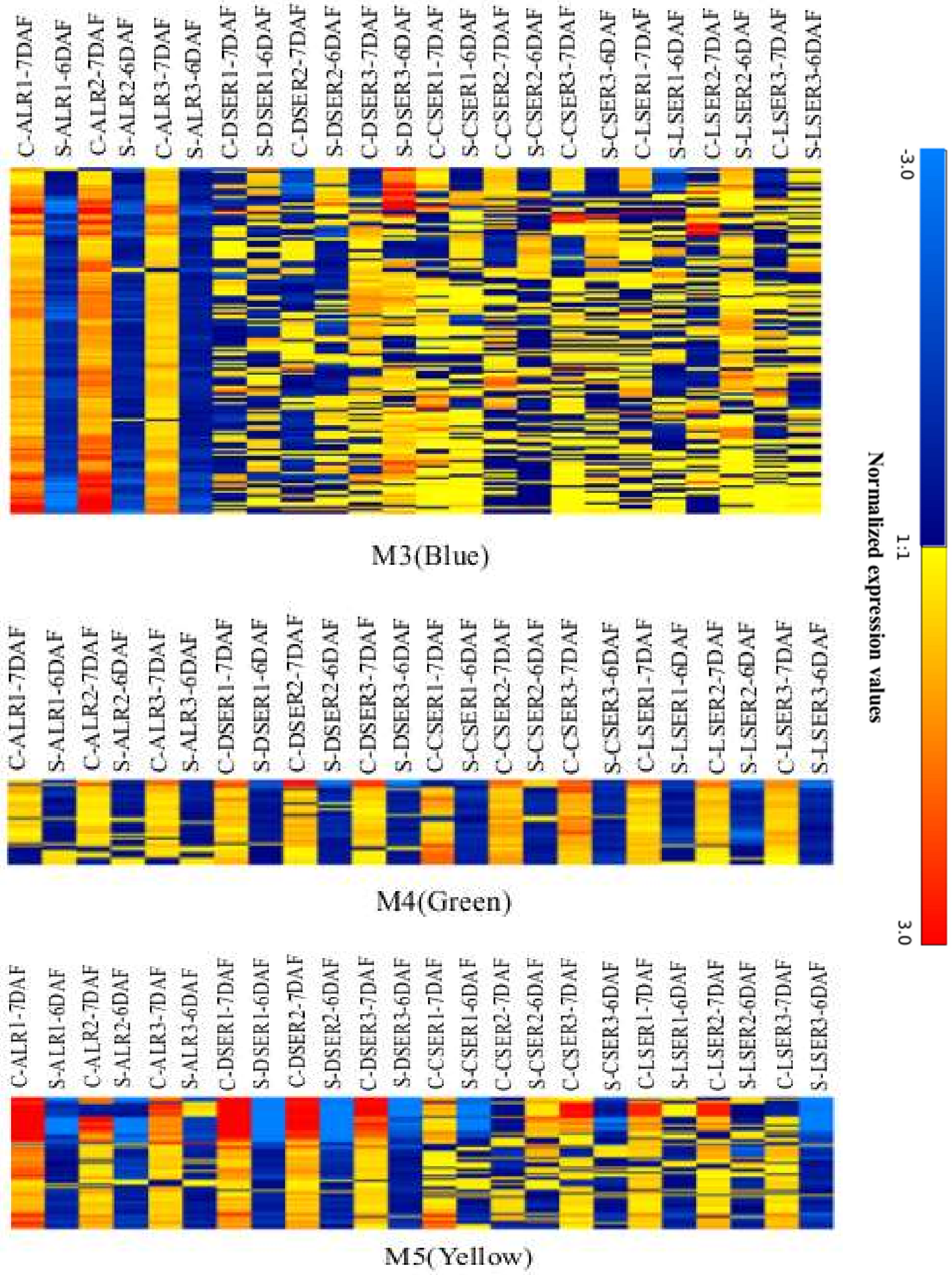

Supplementary Fig.S2 Heat map of co-expressed gene modules.(A) Group of modules upregulated by heat stress across tissues representing modules-M1-turquoise, M2-brown.(B) Group of modules downregulated by heat stress in different tissue types representing modules M3-blue, M4-green and M5-yellow. (log2Expression value +3, red: high -3 blue: low expression)
