# Supplementary material for: Laser Microdissection-Based Tissue-Specific Transcriptome Analysis Reveals a Novel Regulatory Network of Genes Involved in Heat-Induced Grain Chalk in Rice Endosperm
Source: Plant Cell Physiol. 2018 Dec 4;60(3):626–42. doi: 10.1093/pcp/pcy233 (PMC6400107; doi:10.1093/pcp/pcy233)
Supplement: Supplementary Figure S3 [file pcy233_supplementary_figure_s3.pdf]

ISHIMARU ET AL. FIGURE S3

A.GO enrichment of modules upregulated at heat stress condition

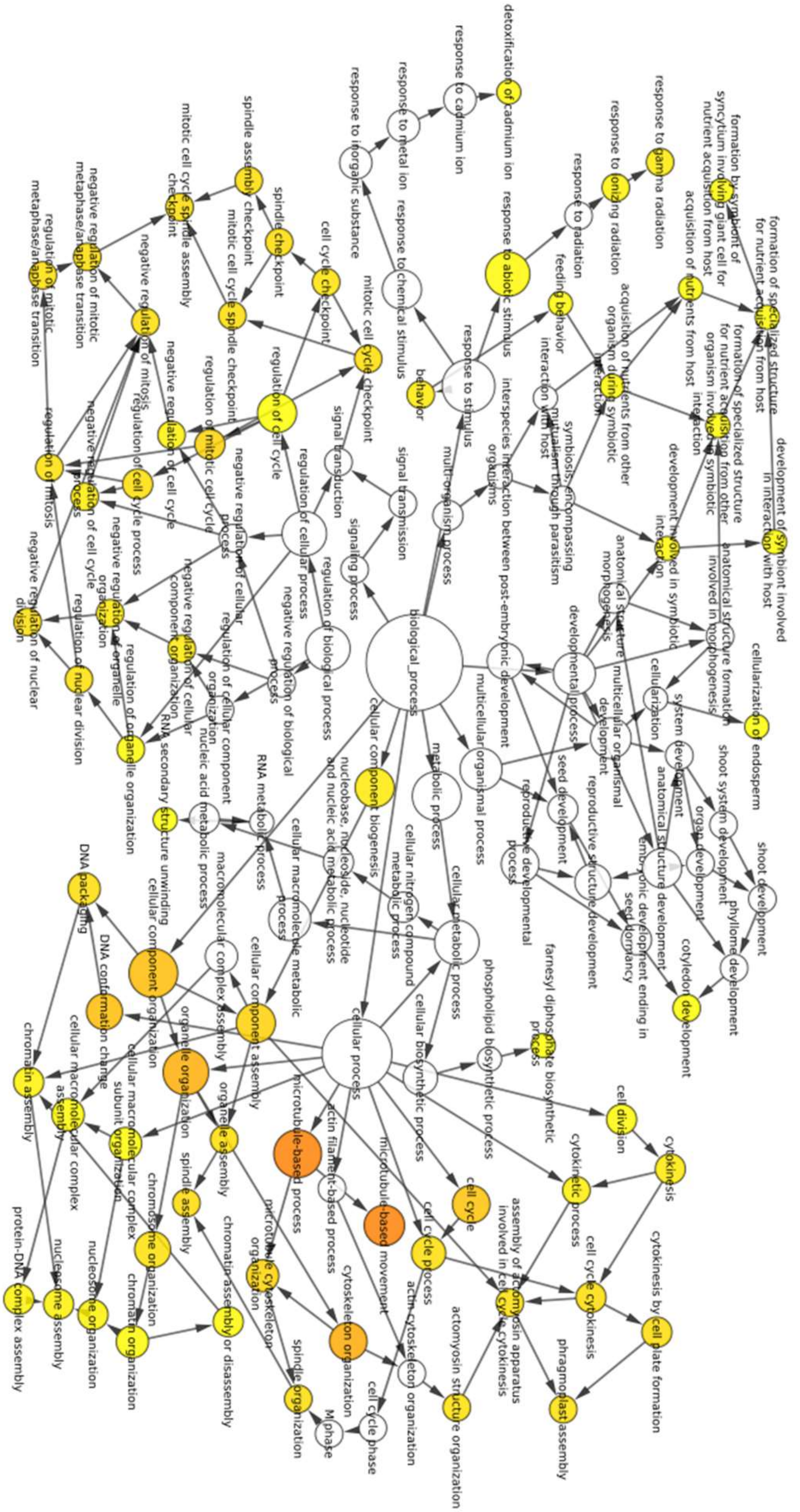

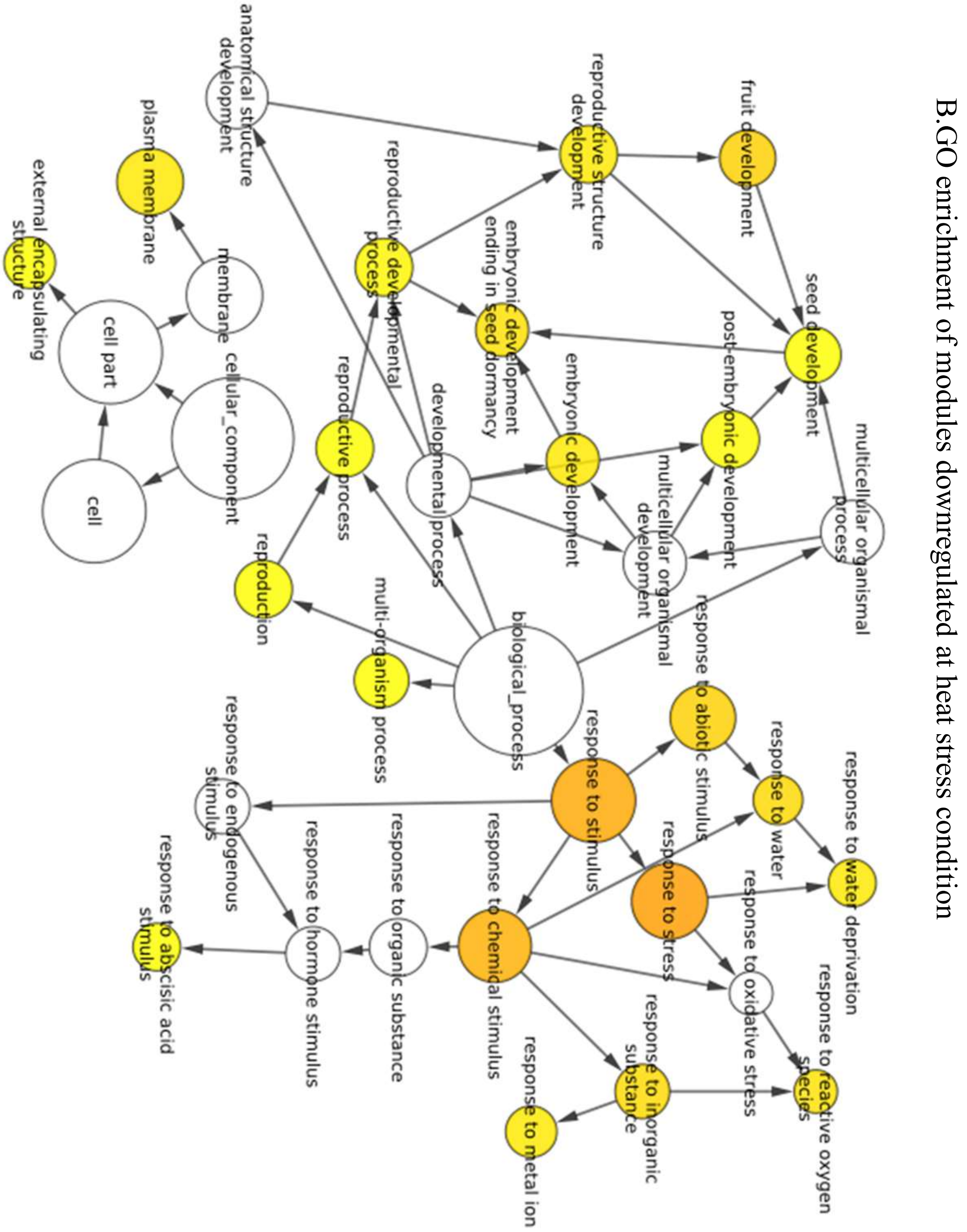

Supplementary Fig.S3 Gene ontology (GO) enrichment analysis of biological process, molecular function and cellular components from upregulated and downregulated selected modules. Node size is proportional to the number of transcripts in each category and colors shaded according to the significance level (white-no significant difference; yellow to orange significant Pvalue 1.00E-2 to <1.00E-7 respectively)(A).GO enrichment of co-expressed genes of modules M1-turquoise, M2-brown.(B)GO enrichment of co-expressed genes of modules M3-blue, M4-green, M5-yellow.
