# Supplementary material for: Laser Microdissection-Based Tissue-Specific Transcriptome Analysis Reveals a Novel Regulatory Network of Genes Involved in Heat-Induced Grain Chalk in Rice Endosperm
Source: Plant Cell Physiol. 2018 Dec 4;60(3):626–42. doi: 10.1093/pcp/pcy233 (PMC6400107; doi:10.1093/pcp/pcy233)
Supplement: Supplementary Table S3 [file pcy233_supplementary_table_s3.pdf]

ISHIMARU ET AL. SUPPLEMENTARY TABLE S3

Supplementary Table S3 Frequency of opaque grains, grain dry weight, and amylose content of *BiP1*-suppressed lines

	Line				
	Vector Control	KD9-1	KD9	KD13	KD12
Dry weight (g grain^{-1})	20.9[a]	14.6[b]	13.6[b]	15.2[b]	15.0[b]
Frequency of opaque grains (%)	0	85.3	79.5	67.5	66.7
Amylose content (%)*	18.4[a]	12.6[b]	13.8[b]	-	-

*15% moisture basis. Only opaque grains were used for KD9-1 and KD9.
Different alphabets indicate significance at 5% level by *Tukey*'s test.
