# Supplementary material for: Laser Microdissection-Based Tissue-Specific Transcriptome Analysis Reveals a Novel Regulatory Network of Genes Involved in Heat-Induced Grain Chalk in Rice Endosperm
Source: Plant Cell Physiol. 2018 Dec 4;60(3):626–42. doi: 10.1093/pcp/pcy233 (PMC6400107; doi:10.1093/pcp/pcy233)
Supplement: Supplementary Table S4 [file pcy233_supplementary_table_s4.pdf]

ISHIMARU ET AL. SUPPLEMENTARY TABLE S4

Supplementary Table S4 Primers used for the generation of *BiP1*-suppressed lines.

Gene name	Accession No.	Primer Sequence	Template
Construction of binary vectors			
BiP1	Os02g0115900	F: AGGATCCATCTGATTCGATTGATTCAAAGG<br>R: AGAGCTCTCTAGACACAGTTCACATGGAAATCAG	AK119653

Restriction sites used for cloning are underlined.
